# Delayed First Language Exposure Negatively Impacts Representation of Small Quantities: Evidence from Deaf and Hard-of-Hearing Children

**DOI:** 10.1162/OPMI.a.32

**Published:** 2025-10-29

**Authors:** Madeline Quam, Emily Carrigan, Kristin Walker, Anna Shusterman, Marie Coppola

**Affiliations:** Department of Psychological Sciences, University of Connecticut; The Connecticut Institute for the Brain and Cognitive Sciences; Department of Psychology, Stony Brook University; Department of Psychology, Wesleyan University; Department of Linguistics, University of Connecticut

**Keywords:** numerical cognition, development, small quantity representation, deaf/hard-of-hearing, language

## Abstract

Most deaf and hard-of-hearing children are born to hearing parents, often delaying exposure to their first language. This negatively influences development of not only language, but also many other aspects of cognition, including exact representations of large quantities. The core knowledge view of numeracy predicts that delays in language exposure should not affect nonverbal representations of small quantities (1–3). This study is the first to investigate effects of language modality (spoken vs. signed) and timing of language experience (early, from birth vs. later) on the representation of small quantities of objects. We adapted the “Mr. Elephant” task (Shusterman et al., 2017) and examined whether children (age 3 to 7 years) succeeded on trials involving quantities 2 and 3. A logistic regression found that Timing and Socioeconomic Status significantly predicted Mr. Elephant performance, while Modality and Age did not. Early-exposed children were more likely to succeed on the task than Later-exposed children. For an exploratory follow-up, two measures of language were added into the analysis: Highest Count, which records children’s recitation of the count list, and Give-a-Number (‘Give-N’), which assesses children’s understanding of the cardinal principle (CP). This logistic regression found that Timing and Give-N performance significantly and independently predicted Mr. Elephant performance, but Socioeconomic Status and Highest Count did not. Children who were CP-knowers were more likely to succeed on Mr. Elephant than non-CP-knowers. These results suggest that the representation of small quantity representations is associated with the timing of children’s language exposure and their knowledge of the cardinal principle.

## INTRODUCTION

Deaf and hard-of-hearing (DHH) students historically are reported to underperform in mathematics compared to typically-hearing peers (Gottardis et al., 2011; Wollman, 1964); however, the reasons behind this have not been fully investigated. It is well documented that early language skills are related to later mathematical success: counting abilities in kindergarten predict later mathematical skills (e.g., Jordan et al., 2009); children’s number word knowledge fully mediates the relationship between general vocabulary and early mathematics achievement (Slusser et al., 2019); and the number talk that primary caregivers engage in with their children predicts (e.g., Levine et al., 2010) and in fact drives (Gibson et al., 2020) children’s knowledge of cardinal number meanings. A working memory system that represents small sets (1–3) is critical for early number development. This foundational number representation system is also related to later numerical skill development, including acquisition of the cardinal principle (Le Corre & Carey, 2007; vanMarle et al., 2018). Small quantity representation has not been thought to depend on language abilities, but this question has not been studied in populations in which language and cognition can be decoupled. Therefore it is unwarranted to assume that the experiences of typically developing individuals are universal. The current study explores this question of the role of language in the small quantity system in the context of DHH children’s highly variable language experiences. We explore the specific impact of children’s understanding of count words and signs, as well as the more general impact of language when it develops on a dramatically different timetable. While studies with populations who receive atypical experience are rare, they provide an important perspective on what experiences critically support even “core” abilities.

### Early Number Knowledge Informs Later Mathematical Skills

Early number knowledge is important for later mathematical achievement (Duncan et al., 2007; Jordan et al., 2009), and difficulty with basic numeracy skills may hinder the learning of more advanced mathematical concepts. The ability to represent small quantities is one of the most foundational numeracy skills; children learn to name small quantities before they learn larger number words (Piantadosi et al., 2014) and in order to learn these labels, children must first be able to represent these quantities. Research suggests that children’s conceptual representation of small quantities of objects does not rely on language (Feigenson & Carey, 2003; Li et al., 2009). However, most of this research focuses on children with typical development experiences and does not consider the effect of later access to a first language on developmental trajectories. Some DHH students’ struggles with mathematics may be traceable to early childhood delays in first language access, postponing the development of number concepts, including the ability to associate number words and/or signs with their corresponding quantities. Indeed, deaf children’s performance on a mathematics achievement test was associated with their timing of access to a first language (i.e., DHH children with at least one signing Deaf parent generally scored better than those with hearing nonsigning parents) as well as their language proficiency, as measured by their American Sign Language (ASL) vocabulary knowledge (Henner et al., 2021). Thus, it is important to understand whether early numerical representations, thought to provide a foundation for number learning in hearing children, are similarly available to support number learning in DHH children, who have variable language experiences.

### Language Should Not Impact the Ability to Represent Small Quantities

Previous literature suggests that one of the cognitive systems for representing quantity–parallel individuation–which is an object tracking system that allows representations of small quantities (1–3), should not rely on language (Carey, 2009). One standard method of assessing small quantity representation is the manual search task in which a child watches 1 to 3 items placed in an opaque box; in test trials the experimenter will covertly put *N*–1 items inside, then allow the child to retrieve the items in the box while timing how long the child searches for the “missing” item (e.g., Feigenson & Carey, 2003).

The small quantity representation system has been shown to be present in a wide range of types of participants and conditions: infants and toddlers (Feigenson et al., 2002; Feigenson & Carey, 2003), adults performing nonverbal tasks (Choo & Franconeri, 2014; Frank et al., 2008; Trick & Pylyshyn, 1994), deaf adults without language input (Spaepen, 2008), adults and children who use languages that do not have counting systems (Butterworth et al., 2008; Frank et al., 2008; Gordon, 2004), and a variety of non-human animals (Gelman & Cordes, 2001) including monkeys (Wood et al., 2008), fish (Piffer et al., 2012), and horses (Uller & Lewis, 2009). Homesigners (deaf adults who have not had access to a linguistic community and who receive little to no linguistic input) perform with high accuracy in match-to-sample tasks (i.e., matching sets of visible disks or repeating exact number of knocks with fists) for small sets (1–3), but show much lower accuracy on trials with larger sets (> 3), compared to individuals with full access to linguistic input (Spaepen et al., 2011). Although homesigners are significantly better at representing small quantities compared to large quantities, they are not perfect, a point which will be addressed later in the discussion. Thus, the development of small quantity representation under atypical experiences, including deafness or delayed exposure to language, is an open question.

### How Does Language Experience Affect Language and Cognition?

Language has widespread effects on many areas of cognitive development, and the effects of altered language experience need to be systematically investigated in order to understand these dynamics. Two key elements of language experience are Modality (i.e., using a signed or spoken language) and Timing (i.e., having access to a first language from birth or later). Deaf and hard-of-hearing children’s language experience varies greatly, for reasons described later in this section. The few recent studies that have systematically examined both language modality and timing generally show that the timing of language experience, but not modality, seems to affect cognitive developmental outcomes (Bandurski & Gałkowski, 2004; Goodwin et al., 2022; Schick et al., 2007). However, no studies with DHH participants specifically investigate how small quantity representation and other foundational skills may be affected by the timing or modality of language exposure.

Language modality—specifically using a spoken or signed language—should not affect core subsystems for representing quantity, including representing approximate number magnitude and small quantity representation. Children acquiring American Sign Language (ASL) from birth from deaf signing parents achieve the same language milestones as children acquiring spoken language (Lillo-Martin & Henner, 2021; Newport & Meier, 1985). Children who acquire ASL from birth also perform similarly to typically-developing hearing children in various areas of cognitive development including executive functioning (Goodwin et al., 2022; M. L. Hall et al., 2017) and theory of mind (Schick et al., 2007). With regard to mathematics, one study of 28 DHH children and 15 hearing children found that, controlling for children’s knowledge of the count list, native-signing DHH children and native English-speaking children showed comparable number knowledge (Secada, 1984). DHH and hearing children who receive sign or spoken language input from birth at home also perform equivalently on verbal, numerical and spatial reasoning tasks (Bandurski & Gałkowski, 2004). Generally, when DHH children are reported to underperform in mathematics related tasks, the participant sample comprises DHH children without early access to language (i.e., later-language-exposed), not DHH children with sign language exposure from birth (Santos & Cordes, 2022). Given that native-signing Deaf children show no evidence of delays, neither deafness itself nor sign language use can explain DHH students’ reported underperformance in mathematics.

Therefore, we must consider other aspects of DHH children’s language experience, specifically when they begin receiving substantial access to their first language, which we refer to as language *Timing*. The vast majority of DHH children are born to hearing parents who do not know a sign language (Mitchell & Karchmer, 2004), delaying full access to their first language. Many parents are encouraged by medical professionals to focus on spoken language acquisition through assistive technology such as hearing aids and cochlear implants, instead of sign language (Mauldin, 2016). Timing of first language access can affect language acquisition, whether the language acquired is signed or spoken. Deaf children acquiring spoken language via cochlear implants show a great deal of variability in spoken language development, even among early-implanted children (Lund, 2016; Niparko et al., 2010). Children with cochlear implants also show delays in number processing and mathematical skills, regardless of the age of implantation (Edwards et al., 2013; Pixner et al., 2014). Further, the language and communicative environments of DHH children limit their opportunities for incidental exposure to linguistic and mathematical symbols, putting these children behind in numerical knowledge before even starting school (Bandurski & Gałkowski, 2004; Pagliaro & Kritzer, 2010).

Timing of first language access affects other aspects of numerical and cognitive development. Children with later exposure to a first language achieve cardinal principle-knower status later than children whose language exposure began at birth, regardless of modality (Shusterman et al., 2022). The benefits of early exposure to ASL for DHH children include better performance on analogical reasoning tasks (Henner et al., 2016) and better theory of mind abilities (Schick et al., 2007). When DHH children have no or delayed exposure to sign language, and also do not receive sufficient access to spoken language, they can be at risk for experiencing long-term language deprivation, which can have significant negative neurological, educational, and developmental consequences (W. C. Hall, 2017).

### Socioeconomic Status and Mathematics

Previous research has documented a well-established relationship between socioeconomic status (SES) and educational outcomes, including mathematics achievement (e.g., Basque & Bouchamma, 2016; Cheadle, 2008; Perry & McConney, 2013), and it is therefore critical to include SES in models of numeracy acquisition. Predictors of preschool numeracy skills include SES, maternal education, and quality of the home learning environment; moreover, the achievement gap for mathematics between higher and lower SES groups was shown to widen from ages 3 to 5 (Anders et al., 2012). SES is also strongly associated with opportunities to learn and subsequent mathematics achievement (Cueto et al., 2014). The effects of SES, specifically parental education, are mediated by acquired language, particularly number language (Slusser et al., 2019). Accordingly, while the current study does not focus on SES as a primary variable, we incorporate it as a relevant covariate.

### Separating Language Experience and Cognitive Maturation

There is substantial evidence from typically-developing children and adults from diverse linguistic backgrounds that language is not involved in small quantity representation. Moreover, a previous study using the same paradigm used here found that preschool-aged children who knew the cardinal principle outperformed children who did not on trials with large set sizes (i.e., 5–7). However, on trials with small sets of 2–3; items, both groups performed at ceiling (Shusterman et al., 2017), suggesting that knowledge of numbers or cardinality is not necessary for this task. This is consistent with the conclusion that language is not involved in similar measures of small quantity representations, such as manual search, on which even very young children succeed (Feigenson et al., 2002). However, in all of these cases, cognitive maturation and linguistic experience are fully confounded. Typically these two variables are linked: adults and older children are more cognitively mature and have years of language experience, whereas young children are still developing cognitively and are in the process of acquiring language. Therefore, it is difficult to disentangle the influences of maturation, perceptual experience, and language experience on the development of the small quantity representation system. DHH children who have delayed exposure to their first language represent a population in which language experience and maturation are not fully confounded (Cheng et al., 2019). That is, we can observe the impact of later access to language in children whose cognitive maturation is typical (at least for abilities that truly do not depend on language input). By comparing abilities in children with typical exposure to a first language (i.e., starting from birth) to children with delayed first language exposure, we can decouple the effects of language from cognitive development and explore the potential effects of language deprivation on cognitive abilities.

Thus, it is not clear whether the foundational skill of representing small quantities develops organically, without any effect of experience, and whether such development would proceed typically even in the absence of typical language exposure or perceptual experience. Non-human animals can track small quantities seemingly without language. Therefore, it would be surprising if language were important for the normal functioning of these representations in humans. Nevertheless, it is possible that this ability is experience-expectant in humans (Greenough et al., 1987). If so, one of the requisite experiences may be typical language exposure, as seems to be the case in so many other domains. Because typical human development expects language, such systems may get disrupted when language input is delayed (Cheng et al., 2019). Since delayed access to a first language can have pervasive and long-lasting effects on language proficiency and neural organization (W. C. Hall, 2017), it is critical to test the effects of deprivation in initial language experiences on the development of small quantity representation. In sum, these considerations raise the question: does timing of first language access affect the ability to track small quantities?

On the one hand, language might not affect small quantity representations, given previous evidence that such representations are operational in non-human animals and in prelinguistic infants (e.g., Feigenson et al., 2002; Feigenson & Carey, 2003; Wood et al., 2008). Furthermore, such representations appear to be intact in a variety of populations without language for exact quantities, as reviewed above (e.g., Spaepen et al., 2011). In a previous study using the same task used in the current study, typically-developing hearing children who did not yet know the Cardinal Principle succeeded, performing nearly at ceiling when they were only required to track small quantities of 2 or 3 (Shusterman et al., 2017). On the other hand, some studies have shown that children who have more advanced number language or who know the Cardinal Principle perform better on non-verbal numerical reasoning tasks, including those that only require representing and comparing small sets (Cheung & Le Corre, 2018; Mix, 1999a). However, those tasks might not have sufficiently invited or motivated children to attend to the quantitative dimensions of the task. For example, Mix’s study used stimuli in which children had to select which picture would match a target with two squares on it – one with two items or one with three; one interpretation is that children who know the cardinal principle are more likely to notice quantitative dimensions. However, in this task, children were not given an explicit reason to match the quantities. By contrast, the current study was designed to create salient reasons for children to attend to the number of items in each target set. The narrative structure of the game, helping “Mr. Elephant” with peanuts stuck in his nose, created a more interactive experience for children who were highly engaged by their role in helping Mr. Elephant. Requiring children to notice if one object was missing, combined with the two initial training trials with two objects, was designed to motivate children to notice whether the set sizes matched. Accordingly, we hypothesized that delays in first language exposure would not be associated with poor performance on tasks requiring representation of small quantities of objects, consistent with the bulk of prior research. Our hypotheses are as follows:

**H1. Language Modality will not affect small quantity representation.**

Since native ASL signers perform similarly to native English users on mathematics and language acquisition, we predict that we will not find a difference in performance for ASL and English groups on a quantity representation task.

**H2. Timing of first language access will not affect small quantity representation.**

Given the well-established findings discussed above showing that a wide variety of populations (e.g., infants, non-human animals, adults without language input) can successfully complete small quantity representation tasks without using language, we predict no difference in performance between children exposed to language from birth and children with delayed access to their first language.

Our primary analysis investigates the effects on small quantity representation of language modality (spoken English vs. ASL) and the timing of first language access (early, i.e., from birth, vs. later, i.e., sometime after birth). Children completed this task as part of a battery of number tasks and other types of tasks. We expected to see decrements in performance due to later timing of language exposure on other tasks, but not in this task, which involves small quantities only. Our exploratory follow-up analysis examines the relationship between children’s small quantity representation and their knowledge of number words.

## METHODS

The data in this study were drawn from a larger project which investigated relations between language experience, mathematical abilities, and other cognitive skills using numerous behavioral tasks and parent-report measures. Typically Give-N and Highest Count were administered first and Mr. Elephant was administered near the end of the testing battery; several tasks, many of which did not include counting, e.g., approximate number representations (Panamath), or in some cases did not even include number, e.g. non-linguistic IQ, cognitive flexibility, were done in between.

### Participants

A total of 153 children, 57 typically hearing and 96 DHH, were recruited from educational programs throughout the United States (Table 1). Their mean age was 5;2 years (*SD* = 11.4 months, range: 3;1–7;5 years); 57% were girls and 43% were boys. Children with known or suspected additional disabilities were excluded. Signed informed consent was obtained from all caregivers and verbal assent was obtained from all participants prior to testing. We obtained a measure of socioeconomic status (SES) with the Barratt Simplified Measure of Social Status which uses parental educational levels and occupational prestige (Barratt, 2006). Possible SES scores ranged from 3 (e.g., the child had one parent with less than a 7th-grade education and not working outside the home) to 66 (e.g., the child had two parents with advanced degrees and high-prestige occupations). Information about the participants’ language experience was also gathered from the background form completed by the caregivers. Participants were categorized into one of four groups based on their language experience (timing of first language exposure and language modality):

**Table 1.** Demographic information.

	**Early English**	**Early ASL**	**Later English**	**Later ASL**
**Total Participants**	57	27	41	28
**Mean Age (years; months)**	4;11	5;4	5;1	5;9
**(*SD*, range)**	(0;9, 3;4–7;4)	(1;2, 3;5–7;4)	(0;10, 3;4–6;7)	(0;11, 3;1–7;5)
**Mean SES** *	54	49	50	37
**(*SD*, range)**	(11, 8–66)	(16, 11–66)	(14, 3–66)	(18, 8–62)
**Gender (% girls)**	63% (36)	67% (18)	49% (20)	50% (14)
**Mean Age of First Language Exposure**	0	0	2;0	3;6
**(*SD*, range)**			(1;6, 0;2–5;7)	(1;1, 1;6–6;3)

* Barratt Simplified Measure of Social Status (Barratt, 2006).

*Early English*: Typically-hearing children who have hearing parents and who began acquiring spoken English from birth

*Early ASL*: DHH children who have at least one DHH signing parent and who began acquiring ASL from birth

*Later English*: DHH children who have hearing parents and who began to acquire spoken English in early childhood via hearing technology (e.g., cochlear implant, hearing aid) and attended an oral program that emphasized listening/spoken language

*Later ASL*: DHH children who have hearing parents and who began to acquire ASL in an educational program that used ASL as the primary language of instruction

**Figure 1.**
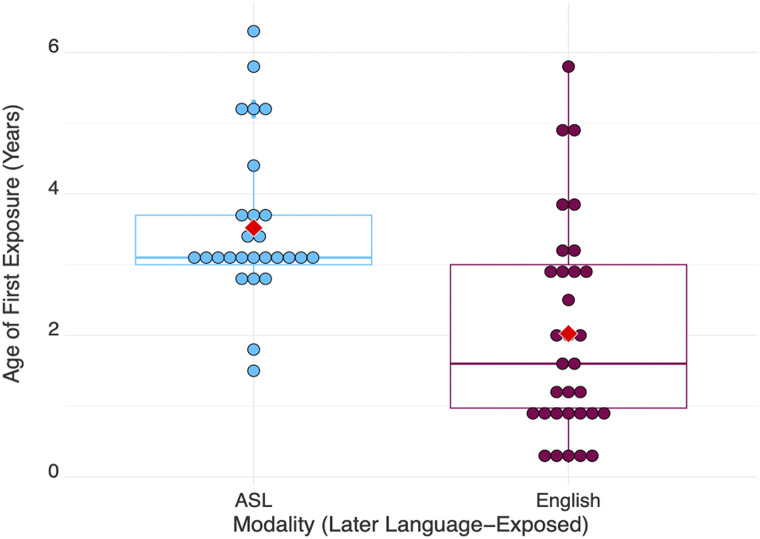
Age of first exposure to language for DHH children exposed to ASL and English later (i.e., some time after birth. All early-exposed children’s first exposure to language is 0 (i.e., starting from birth) and are not included in the graph. On average, children were first exposed to ASL at 3;6 years old (*SD* = 1;1) and to English at 2;0 years old (*SD* = 1;6). Each dot represents one child. The red diamond represents the mean.

We recognize that these categories cannot perfectly capture every aspect of children’s language experiences. However, we decided to use Language Timing as a binary variable (Early vs. Later) because we are confident that children in the Later Language groups had less cumulative exposure to language due to their limited access to language compared to children in the Early Language groups, who had full, uninterrupted access. For children in the Later English group, we recorded ‘age of first language exposure’ as the age when the child received assistive hearing technology for children acquiring spoken English. For children in the Later ASL group, we recorded the age at which the child began attending a program or school that used ASL (Figure 1). While this measurement of first substantial access to language is not perfect, it is important to note that all children in the Later Language group experienced later and/or reduced access to language to some degree. See Carrigan and Coppola (2020) for further discussion.

### Materials

To measure small quantity representation we adapted the “Mr. Elephant” task, which was created by Shusterman et al. (2017) as an alternative to manual search tasks, and which allowed the child to provide a clear, unambiguous response.

**Figure 2.**
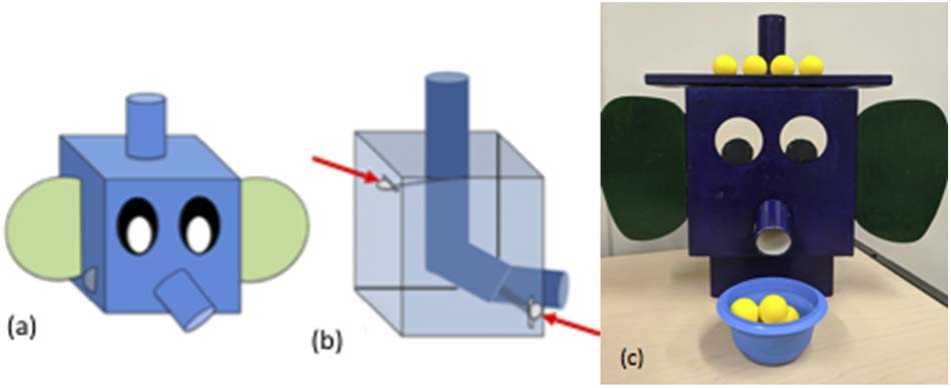
Schematic diagrams of the exterior (a) and interior (b) of Mr. Elephant, and a photo (c) (Shusterman et al., 2017).

Mr. Elephant was a custom-built wooden model made to look like the head of an elephant (Figure 2a). A cylindrical chute on the top of Mr. Elephant’s head and another chute that came through the trunk were connected by an internal tube (Figure 2b). Small yellow balls (“peanuts”) were placed inside the tube on the top of the head and could be either trapped or released from the trunk. A lever on the backside of Mr. Elephant controlled a small plastic door near the top (indicated by red arrows in Figure 2b), trapping balls by sliding out to cover the chute. Another lever behind Mr. Elephant’s ear controlled a small plastic door near Mr. Elephant’s trunk. This lever released balls by displacing the plastic door, allowing balls to continue rolling out of Mr. Elephant’s trunk, into a small bowl, which caught the balls.

### Procedure

Parents filled out questionnaires regarding demographic information. For all behavioral tasks reported, each child was tested individually, in their preferred language (ASL or spoken English) by either a deaf, fluent ASL user or a native English speaker. The instructions were carefully developed to be comparable across the two languages by a team of deaf and hearing users of English and ASL. All interactions were video recorded. Children were tested in a distraction-free environment, most at school and a small portion at home or in the laboratory. One of the experimenters who collected the data knew the hypotheses of the study, but the others were not aware of them. All experimenters were aware of children’s language modality and the timing of their language exposure.

At the beginning of each trial, the set of balls to be dropped was placed on top of Mr. Elephant and was visible to the child (as in Figure 2c). Then the experimenter sequentially dropped *N* (2–7) balls into Mr. Elephant; either *N* or *N*–1 balls exited via his “trunk”. Children indicated whether any balls remained inside. One experimenter provided instructions to the child, while another (the ‘facilitator’) stood behind Mr. Elephant to operate the levers and add balls when necessary. The child was seated in front of and facing Mr. Elephant, so they could see the balls enter and exit. The experimenter ensured the child was paying attention throughout this procedure and could not see the levers being operated by the facilitator. The task was conducted in three phases: demonstration, training, and test trials. The task aimed to see if the child noticed a change in the number of balls that went in and came out of Mr. Elephant and was able to accurately track the quantity of objects. In the demonstration phase, the experimenter and facilitator introduced Mr. Elephant to the child and demonstrated how Mr. Elephant worked. The facilitator operating Mr. Elephant first dropped a single ball into the chute on top of Mr. Elephant’s head, and allowed it to be released. When the ball was released, the experimenter congratulated Mr. Elephant (e.g., “Good job!”). Then, the facilitator demonstrated a single ball being inserted and getting “stuck.” Here, the experimenter told the child that the ball was stuck in the trunk and that Mr. Elephant had to “sneeze” it out. The ball was released after the child either signed SNEEZE or said “achoo”. Children were not instructed or encouraged to count during this task, and the instructions did not include words like “number” and “count”. On each trial, the target number of balls was lined up in a row on top of the apparatus, and the balls were dropped in sequentially.

The training phase consisted of two trials where a single ball was either stuck or released and the child was asked whether they should tell Mr. Elephant “good job” (i.e., there were no balls left inside Mr. Elephant’s trunk) or “help him sneeze” (i.e., a ball was still stuck inside). In training trial 1, the ball was dropped and immediately released. If the child responded to this trial indicating that they recognized all the balls that entered had exited Mr. Elephant, the experimenter agreed. However, if the child indicated that they thought another ball was still inside Mr. Elephant, the experimenter responded by telling the child that Mr. Elephant was “all done”. In training trial 2, the facilitator dropped and trapped one ball. Again, the child was asked whether they thought a ball was still inside Mr. Elephant. If the child responded “sneeze” or otherwise indicated that they knew a ball was still inside, the experimenter agreed. If the child responded “good job” or indicated that they thought all the balls had exited, the experimenter told the child that there was a ball stuck in Mr. Elephant’s trunk and that they needed to help him sneeze. If the child failed either training trial, the experimenter repeated them until the child succeeded on each trial one time. For the six test trials, the facilitator dropped and released the number of balls as indicated by the protocol sheet (see Appendix for trials). For example, in the first trial, the facilitator dropped five balls, then released four, trapping one ball. After releasing the target number of balls, the experimenter asked the child the same question (e.g., tell Mr. Elephant good job or help him sneeze?) and provided the same feedback (e.g., “that’s right”, “actually Mr. Elephant is all done” or “we need to help him sneeze”) as in the training phase.

We were interested in small quantity representation, so we entered into the analyses whether children succeeded on the two trials involving quantities 2 and 3 (2 in, 2 out; and 3 in, 2 out). Since there were only two small quantity trials, we were unable to distinguish between children who answered one trial successfully (50% correct) and children who answered no trials correctly (0% correct) as neither pattern indicates above-chance performance. Therefore, we treated performance as binary: the child either got both trials correct or did not get both correct.

A total of 181 children participated in the Mr. Elephant task; 28 participants were excluded for one or more of the following reasons: (a) the child did not complete both small quantity trials (8 children), (b) equipment malfunction or experimenter error (e.g., a ball got stuck on a trial where all balls were supposed to come out; 7 children), or (c) the child did not understand the task, as noted by experimenters or coders (14 children). In order to determine whether a child understood the task, we looked at a variety of behaviors (many of which the 14 children did more than one) including: repeatedly failing the training trials (maximum of 4 times per trial; 4 children), not completing both training trials (8 children), answering the same response every time (12 children responded “sneeze” on every trial, 1 child responded “finished” on every trial), and/or not responding or engaging with the task (3 children).

## RESULTS

**Figure 3.**
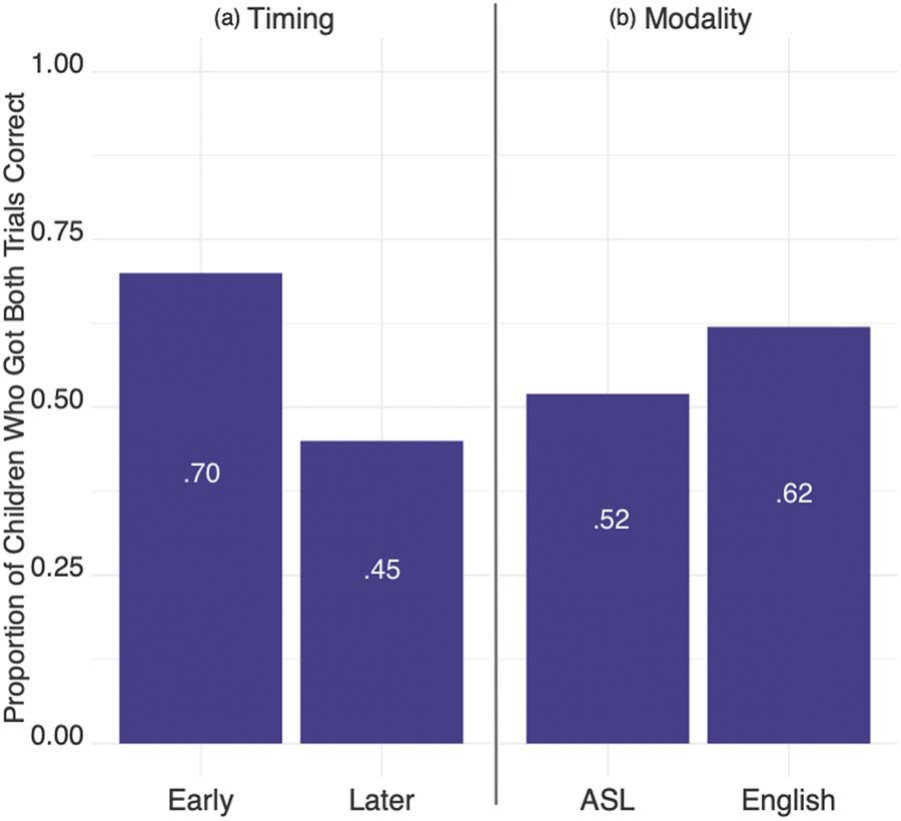
(a) Timing of language exposure is a significant predictor for performance on Mr. Elephant, but (b) Modality of language is not. More children in the Early Language group succeeded on the Mr. Elephant trials compared to the Later Language group. There was no significant difference between the English and ASL groups (*X*^2^(1, *N* = 153) = 1.605, *p* > 0.2).

We ran a logistic regression including Timing, Modality, SES and Age as predictors of performance on the Mr. Elephant small quantity trials (i.e., 2 and 3 items) (Table 2).^1^ The logistic regression found that Timing (*β* = 0.95, *p* = 0.01) and SES (*β* = 0.029, *p* = 0.028) significantly predicted the likelihood of answering the small quantity trials correctly, while Modality and Age did not (Figure 3). Children in the Early Language group were 2.58 times more likely to get both trials correct than children in the Later Language group. Additionally, for every 1 point increase in SES, children were 1.03 times more likely to succeed on the task. Models including an interaction between Timing and Modality (AIC = 191.83) or Timing and Age (AIC = 190.63) fit the data less well than the reported model (AIC = 189.83). An ANOVA likelihood ratio test found no significant difference between the reported model and either a model with an interaction between Timing and Modality (*X*^2^(1) = 0.0035, **p* > 0.95*) or a model with an interaction between Timing and Age (*X*^2^(1) = 1.198, **p* > 0.27*).

**Table 2.** Logistic regression results. Timing of language exposure and SES were significant predictors of performance on the Mr. Elephant task.

**Logistic Regression Results**	
	Mr. Elephant Performance *β* (SE)
**Timing (Early)**	0.949^*^ **(0.370)**
**Socioeconomic Status**	0.029^*^ **(0.013)**
Modality (English)	0.072 (0.397)
Age	0.350 (0.207)
Constant	−3.346* (1.405)
Observations	145
Akaike Inf. Crit.	189.829
Residual Deviance	179.83

*Note:* *p < 0.05.

Because SES was a significant predictor of Mr. Elephant performance, we used *t*-tests to compare SES scores between groups and found major disparities. In terms of Modality, the SES of the children in the English group (*M* = 52, *SD* = 12.6) was higher than that of the ASL group (*M* = 42, *SD* = 18.1), (*t*(79) = −3.24, *p* < 0.002). Similarly, with regard to Timing, children in the Early-exposed group (*M* = 52, *SD* = 13.3) had higher SES scores than the Later-exposed group (*M* = 44, *SD* = 16.8), (*t*(125) = 3.12, *p* = 0.002). A model with just SES, Age and Modality, (excluding Timing) fit the data less well (AIC = 194.57) than the reported model and an ANOVA likelihood ratio test found a significant difference between the reported model and the model excluding Timing (*X*^2^(1) = 6.74, **p* < 0.01*), indicating that Timing explains variability in Mr. Elephant performance over and above any variability explained by SES.

### Discussion

These findings show that Timing of access to a first language, but not Modality of language, predict children’s ability to represent small quantities. This finding is unexpected, since most previous literature suggests that the small quantity representation system is independent from language. However, there is some evidence that small quantity representation may be influenced by language. Mix (1999a) found that children’s small quantity representations could be impacted when matching sets with surface level differences (e.g., size, shape, array dispersal), but children with a strong sense of the cardinal principle tended to perform better at matching dissimilar sets compared to non-cardinal principle knowers. Additionally, Cheung and Le Corre (2018) found that cardinal principle knowers were more accurate at comparing small sets than non-cardinal principle knowers.

The current findings could be explained in two ways. First, it is possible that the same mechanisms driving the effects of language on small-number representation in Cheung and Le Corre (2018) and Mix (1999a) operate with Mr. Elephant. However, this would be surprising given that, in a non-Deaf sample, children who were not cardinal principle knowers were at ceiling, with nearly perfect performance, on Mr. Elephant (Shusterman et al., 2017). Note that the outcome variables were analyzed differently; Shusterman et al. (2017) reported percentage of trials correct whereas the current study treated the outcome as binary, children either got both trials correct or they did not. Second, it is possible that children who experience a delay in first-language exposure proceed on a different developmental path than children with typical native language exposure from birth, and this results in an aberrant, and less functional, small number representation system. We return to these possibilities in the general discussion.

The effect of language timing on performance on the Mr. Elephant small quantity trials indicated that language experience might predict small quantity representation, contrary to most previous evidence. Accordingly, our exploratory follow-up analysis further investigated what exactly about later exposure to language may have influenced children’s performance on the Mr. Elephant small quantity trials. Given that two previous studies (Cheung & Le Corre, 2018; Mix, 1999a) found evidence that cardinal principle knowledge may be associated with small quantity representation, we explore whether our participants’ cardinal principle knowledge might be related to their performance on the Mr. Elephant task.

## EXPLORATORY FOLLOW-UP ANALYSES

Given the surprising results of our primary analysis suggesting that the timing of children’s language exposure relates to small quantity representation, we wanted to explore what in particular about Language Timing might direct this performance pattern. Previous literature has documented that small quantity representation abilities contribute to children’s achievement of the cardinal principle (e.g., Negen & Sarnecka, 2009; vanMarle et al., 2018; for discussion of enriched parallel individuation see Le Corre & Carey, 2007; see also Carey et al., 2017 and Carey & Barner, 2019, for negative arguments that other systems are unlikely to support the acquisition of cardinality). Across diverse populations and ages, children first learn the words for small quantities and only subsequently pass tests of the cardinal principle (Piantodosi et al., 2014; Sarnecka et al., 2007; Shusterman et al., 2022). It is therefore likely that, under typical developmental conditions, the foundational small quantity number representations help scaffold learning of the cardinal principle (CP). Following this line of argument, we would expect that performance on the Mr. Elephant task would predict later performance on an assessment of cardinal principal knowledge (e.g., Give-a-Number). However, finding that language is related to our small quantity representation task also led us to consider the potential for a bidirectional influence between quantity representation and number word knowledge. Previous evidence suggests a reciprocal relationship between symbolic and nonsymbolic numerical processing (see Goffin & Ansari, 2019 for a review) and a previous study with Mr. Elephant involving representing *larger* quantities (5–7) found that CP-knowers performed significantly better compared to non-CP-knowers (Shusterman et al., 2017).

We therefore decided to evaluate whether children’s number language is associated with their small quantity representation in this task and population. In order to do so we included two different language measures that were completed by children close in time to when they did the Mr. Elephant task: (1) Highest Count, which records how high the child can correctly recite the count list (in English or ASL), and is a proxy measure for number-word input that the child has been exposed to, and (2) Give-a-Number (‘Give-N’), which indexes achievement of the cardinal principle and assesses whether children know the exact quantities associated with specific number words/signs. The fact that Language Timing predicted performance on even small quantity (2–3 objects) trials for Mr. Elephant suggests that language may be important in shaping the small quantity representation system even in quantity representation tasks. Indeed, delayed numeracy development (as assessed by Give-N performance) is fully explained by delayed language development in DHH children (Shusterman et al., 2022), so it is possible that differential knowledge of number word meanings for Early- and Later-exposed children may explain why Language Timing predicted performance on the Mr. Elephant task. To summarize, our questions in these follow-up analyses are: (i) Is there an association between small quantity representation and understanding the cardinal principle?, (ii) Does knowing number words in a sequence (Highest Count) and/or knowing the cardinal principle (Give-N performance) predict children’s performance on small quantity representation (Mr. Elephant)?, and (iii) Does language experience (specifically Timing) explain variance in Mr. Elephant performance beyond cardinal principle knowledge?

### Methods

#### Participants.

We analyzed data from the same children who participated in the original analysis. Eight children (4 Early English, 1 Early ASL, 1 Later English, and 2 Later ASL) did not complete the Give-a-Number and Highest Count tasks in addition to the Mr. Elephant task. Of the remaining 145 participants, 53 were in the Early English group, 26 Early ASL, 40 Later English, and 26 Later ASL.

##### Procedure

###### Give-a-Number.

**Figure 4.**
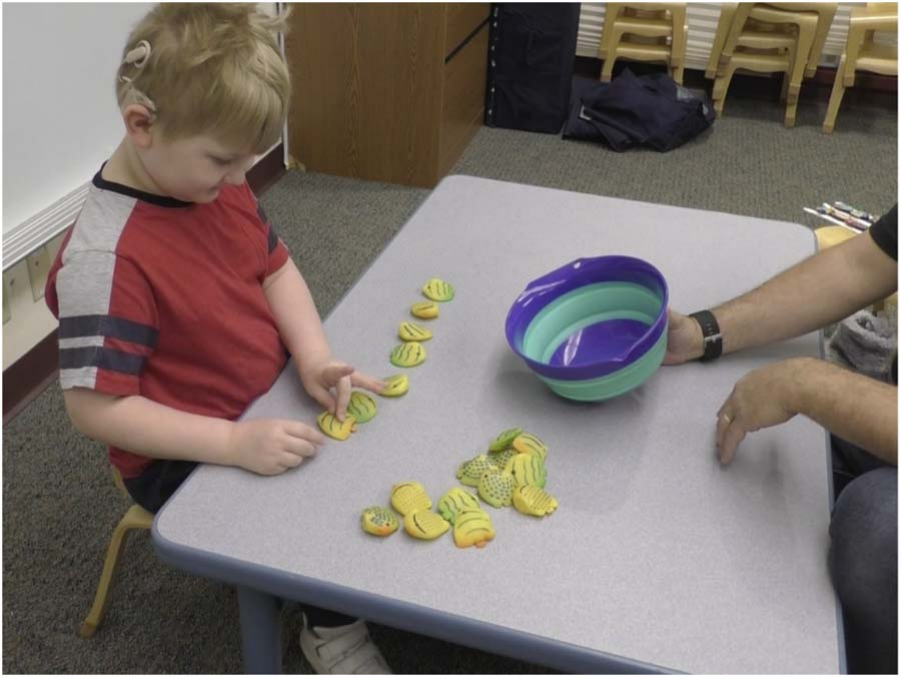
Example of a participant completing the Give-a-Number task.

This task (Give-N) used one large bowl and twenty rubber toy fish (Figure 4). The experimenter presented each child with the 20 fish and placed the bowl on the table between them. On each trial, the experimenter asked the child to put [N] *(e.g., the target number of)* fish in the bowl. If the child spontaneously counted while generating the set, the experimenter looked away to avoid the possibility that the child would rely on the experimenter for cues.

After the child stopped putting fish in the bowl, the experimenter took the fish out of the bowl, lined them up on the table, and asked, “Is that [N]?” If the child responded “No,” the experimenter asked the child to fix it to make it [N]. If the child responded “Yes,” the experimenter asked “Can you count and make sure there are [N]?” The experimenter provided positive feedback regardless of the child’s accuracy. Once the child finished the “count and make sure” procedure, the experimenter moved on to the next target value. Instead of using the typical titration method (Schaeffer et al., 1974; Wynn, 1990), we tested all quantities 1 through 6 three times each in a fixed random order.

For the current analysis, we treated Give-N performance as binary: CP-knowers and non-CP-knowers. Children who produced accurate sets for both quantities 5 and 6 on at least 2 out of 3 trials were classified as “CP-knowers.” Children who did not meet this criterion were classified as non-CP-knowers.

###### Highest Count.

The elicited counting task followed a similar protocol to Sarnecka and Carey’s (2008) sequence task that asked children to count to 10. Here we first asked children to count to 10; if they succeeded, we then asked them to count to 20. We recorded the highest number reached without errors in either set (counting to 10 or counting to 20). The child’s highest count was used if they counted more than once.

### Results

To examine whether Mr. Elephant performance predicted Give-N performance, as we expected, we conducted a logistic regression that included Mr. Elephant, Timing, SES, and Age^2^ as predictors of CP-knower status. The model results showed that both Mr. Elephant performance (*β* = 1.660, *p* = 0.004) and Age (*β* = 1.472, *p* < 0.001) significantly predicted CP-knower status. Children who succeeded on the Mr. Elephant small quantity trials were 5.24 times more likely to be CP-knowers. An ANOVA likelihood ratio test showed that a model with Mr. Elephant was significant compared to a model without (*X*^2^(1) = 9.182, *p* < 0.003).

**Figure 5.**
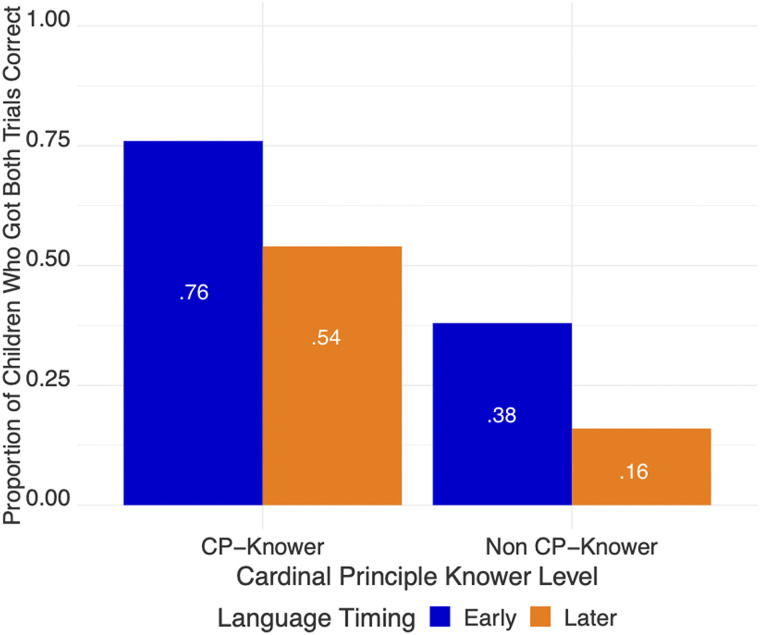
Give-N performance and Language Timing independently predicted Mr. Elephant performance. Children who were CP-knowers were more likely to succeed than non CP-knowers on the small quantity representation task in Mr. Elephant. Additionally, children with early language exposure were more likely than children with later language exposure to succeed on the small quantity representation task. Note that most of the children were CP-knowers (83%).

Next we re-ran the original model of Mr. Elephant performance (that was used in the first study) using the participants who had also completed the Give-N and Highest Count tasks (reducing the sample from 153 to 145 participants), and found the same results: Language Timing (*β* = 0.85, *p* < 0.03) and SES (*β* = 0.026, *p* < 0.05) were significant predictors of Mr. Elephant performance, while Age and Modality were not. We then added children’s Highest Count and Give-N performance as additional predictors. In our sample, 120 children were cardinal principle knowers and 25 were not. A logistic regression model with Timing of language exposure, SES, Give-N, and Highest Count found that Timing (*β* = 0.83, *p* < 0.04) and Give-N (*β* = 1.76, *p* < 0.005) were significant predictors of Mr. Elephant performance (Table 3). Children in the Early Language group were 2.29 times more likely to succeed on Mr. Elephant than children in the Later Language group. Children who were CP-knowers were 5.8 times more likely to succeed on Mr. Elephant than children who were non-CP-knowers (Figure 5). Note that the effect of SES found in the first model disappeared once Give-N and Highest Count were added into the current model.

**Table 3.** Logistic regression results. Timing of language exposure and Give-N performance significantly predicted Mr. Elephant performance.

**Logistic Regression Results**	
	Mr. Elephant Performance *β* (SE)
**Timing (Early)**	0.828^*^ **(0.396)**
Socioeconomic Status	0.018 (0.013)
**Give-N (CP-Knower)**	1.757^**^ **(0.614)**
Highest Count	−0.011 (0.044)
Constant	−2.191* (0.890)
Observations	137
Akaike Inf. Crit.	173.082
Residual Deviance	163.08

*Note:* *p < 0.05; **p < 0.01.

Additionally, this model (AIC = 173.08) fit the data better than the original model (AIC = 182.39) run with the same subset of participants. An ANOVA likelihood ratio test found that the addition of Give-N and Highest Count made the model fit the data significantly better than the model without these two predictors reported above (*X*^2^(2) = 9.41, **p* < 0.01*), indicating that this exploratory model is a better fit than the original model. Other models including Modality and Age as additional predictors similarly found significant contributions of Timing of language exposure and Give-N, but fit less well (AIC = 176.97) than the reported model. An ANOVA likelihood ratio test showed no significant difference between models including or excluding Highest Count (*X*^2^(1) = 0.06, *p* > 0.8), suggesting that Highest Count did not independently explain any of the variance in Mr. Elephant performance. Models that included an interaction between Give-N and Timing or Give-N and Age did not find a significant interaction between the two variables and fit less well (Timing * Give-N: AIC = 176.93; Give-N * Age: AIC = 176.94).

The reported model indicates that Give-N, along with Timing of language exposure, but not Highest Count, predicted performance on Mr. Elephant. Thus, simply being able to recite the number words in sequence did not influence small quantity representation, but understanding the cardinal principle as measured by Give-N may facilitate small quantity representation. Notably, and consistent with prior research, these measures are often discrepant: for example, one child in the Later ASL group was able to count to 20 when tested on Highest Count, but could only create accurate sets of fish up to 3 in the Give-N task. Being able to recite the count list correctly does not necessarily mean the child can ascribe meaning to each of the number words (e.g., Le Corre et al., 2006; Wynn, 1990, 1992).

### Discussion

These findings suggest that both Timing of language exposure and knowledge of the cardinal principle relate to performance on Mr. Elephant small quantity trials. On average, later access to language, whether it be spoken (Shusterman et al., 2022) or signed (Coppola & Walker, 2025) negatively impacts children’s acquisition of number words and their meanings. The previous findings point to a relation between the timing of language exposure and acquiring the cardinal principle; however, the relation to small quantity representation is new and surprising. Critically, although learning the cardinal principle was related to better performance on the small quantity representation task, it did not replace the advantage afforded by early access to language. Therefore, language, both general timing of first exposure to language as well as specifically understanding the meanings of number words, is important for the Mr. Elephant task, a measure of small quantity representation skills.

## GENERAL DISCUSSION

This study produced unexpected findings that language experience, and factors specifically related to delayed language exposure, may actually play a role in children’s small quantity representation abilities, not cognitive maturation. Our primary analysis found that the timing of first access to language predicted success on a small quantity representation task, while language modality (signed or spoken) had no effect. These results prompted exploratory follow-up analyses, which found that, in addition to timing of language exposure, understanding the cardinal principle predicted success on the small quantity representation task, but count sequence knowledge and general cognitive maturation did not.

While these results are correlational, they nevertheless suggest that language development, small quantity representation, and cardinal principle knowledge are interrelated. In light of these findings, further studies exploring the general relation between the onset of language exposure and small quantity representation are needed. It is still unclear exactly how timing of language exposure and knowing the cardinal principle influence small quantity representation abilities. It is also unclear exactly which aspects of language exposure or input are most related to cardinal principle knowledge in deaf and hard-of-hearing children. Future investigation will likely help us better understand current theories about the development of systems of number representation and the mechanisms involved in representing small exact sets in all children.

### Using Counting as a Strategy

While the Mr. Elephant task could be solved using counting as a strategy, it seems unlikely that the children in this study used counting, especially on trials with only 2 and 3 balls. We coded spontaneous counting (e.g., speaking or signing the number words, pointing systematically to each of the objects in the set) in another task that was a part of the larger project; however, we did not observe overt counting behavior from children during the Mr. Elephant task. Recent research shows that children do not count even on tasks in which it would be beneficial to count, unless explicitly trained to count beforehand (Posid & Cordes, 2018), and even when children demonstrate mastery of counting, they do not always use counting to solve non-verbal quantity tasks (Shusterman et al., 2017). However, hearing children with math difficulties tend to count all items (as measured with eye-tracking) when presented with small quantities (1–4) compared to hearing children with typical math skills who rely more on subitizing abilities (Schindler et al., 2020).

While language-based counting does not seem very likely in the current Mr. Elephant task, children might have used nonlinguistic, unobservable counting. Nonlinguistic counting has been demonstrated in Alex the African gray parrot, who was able to identify sets of objects (1–6) with 83% accuracy (Pepperberg, 1994), but this capacity has not been documented in humans.

Future steps should investigate how children solve tasks like Mr. Elephant: using the object tracking system, as we hypothesized; subitizing (which would require recruiting some symbolic number knowledge); or some kind of nonlinguistic counting. Further, it would be of interest to know whether early and late language exposure affects how children approach tasks where counting or symbolic number representations are, in principle, not needed. An electrophysiological study (using methods similar to Hyde et al., 2017) might help to triangulate whether children can use pure small quantity representations, without a language component, during their online processing in these tasks.

### No Effect of Age on Small Quantity Representation Abilities

It was somewhat puzzling that children’s age had no effect on their Mr. Elephant performance. Previous research also suggests that working memory, a skill likely recruited in the Mr. Elephant task, also improves with age and development (e.g., Kibbe & Applin, 2022; Pailian et al., 2016). Since Give-N performance improves with age and also relates to Mr. Elephant performance, the fact that age did not predict Mr. Elephant performance was unexpected. This small quantity representation task may have been very simple for children with typical language experience in this age range, lessening the likelihood of age being a significant factor for children in the Early Language group. Delayed language exposure, however, can have persistent and pervasive negative effects on many aspects of cognitive development (W. C. Hall, 2017), so for the children in the Later Language group, this Timing effect likely overshadowed any possible age or maturational pattern we might see in children whose language experience begins at birth.

### Is Language Involved in the Mr. Elephant Task?

It is unclear whether Mr. Elephant is a completely nonlinguistic task. This task is methodologically similar to the classic cracker choice study (Feigenson et al., 2002) or manual search tasks (Feigenson & Carey, 2003) and conceptually similar to the nuts-in-a-can task (Frank et al., 2008). Children were not prompted to count the balls, and since they only have to track two to three balls at a time, we assumed it was possible to succeed without counting. The language used in the task instructions was relatively simple, and was produced by fluent users with extensive experience working with children. Regardless, it may have been challenging for children in the later-exposed groups to understand. Because children completed two training trials (involving just 1 ball) before the test trials, we assumed that they understood the task expectations. Of the 28 participants who were excluded from analysis, 13 were excluded (8 from the Later Language group, 5 from the Early Language group) because they did not understand the Mr. Elephant task. For example, a child who answered “stuck” on every test trial and answered training trial 1 (see Appendix) incorrectly four times was excluded; based on their poor practice trial performance, we could not be sure that this child understood the task. Due to our careful evaluation of training trial performance, the remaining children included in the analysis met the criteria for understanding the task, regardless of Language Timing status. Nevertheless, children were required to use language to respond, which could have affected their performance.

Research with homesigners (deaf adults who have not had access to a linguistic community) has found that they struggle even in an experiential false belief task whose methodology does not rely on understanding a story (Gagne & Coppola, 2017). While the false belief task itself was minimally linguistic, participants were required to produce an explicit non-linguistic prediction (i.e., circling the picture of the item they thought the confederate would choose); this cognitive load may have prevented homesigners from demonstrating their knowledge. More broadly, it is plausible that requiring an explicit response on an otherwise non-linguistic task could be linked to language abilities, putting language-delayed children at a disadvantage. For example, DHH children with delayed access to language performed significantly worse on a nonlinguistic reasoning task (picture similarities) compared to DHH children with full access to language (Quam & Coppola, 2023). Future research could explore using eye-gaze patterns or neuroimaging techniques to eliminate language from a small quantity representation task altogether.

### Resolving Discrepancies Between Current Findings and Previous Research

Another unresolved question is why young infants, as well as adult homesigners who do not have access to linguistic input in their environment, are able to represent small quantities (Feigenson et al., 2002; Spaepen, 2008), but the children in the current study who had delayed or interrupted access to language had trouble with it. As noted in the introduction, the small quantity system appears to be online early, perhaps even from birth (Martin et al., 2022). Thus, the failure of older children to engage this system in the Mr. Elephant task is surprising. Access to language is a normative, experience-expectant developmental process; thus, it is possible that being deprived of this experience alters the functionality of core systems. We consider possible effects of late language on the small quantity representation system in more detail below.

Homesigners, unlike typically developing infants, but like the participants in our study, experience atypical language input. While the adult homesigners performed better well on some tasks, this was not the case when objects were not in continuous view – similar to the Mr. Elephant task – or were not objects but rather a series of knocks that they had to replicate. Under those conditions, the task became more challenging, even for the small quantities (Spaepen et al., 2011). Adult homesigners have spent a lot more time interacting with the world and using numbers, whereas the children in the Later Language group had much less experience (at most seven years), and adults are better than children at attending to more objects (Trick et al., 2005). Therefore, the non-ceiling performance of adult homesigners in previous studies, and the poor performance of the late-language children in this study, might be more similar than it seems at first glance: it is possible that the performance of both groups on working memory tasks with small numbers of objects is negatively impacted by their late exposure to language.

Additionally, the current results do not align with Shusterman et al.’s (2017) finding that both subset knowers and cardinal principle knowers performed at ceiling on Mr. Elephant small quantity trials. The current study’s findings that children comparatively underperformed is unexpected, especially since children as young as 12- to 14-months old should be able to keep representations of up to three objects in their working memory (e.g., Feigenson & Carey, 2003). Here, we posit a few reasons to explain this discrepancy in performance. First, all of the participants in Shusterman et al. (2017) were recruited from a previous study in which they had to complete number related games (e.g., Give-N, Fast Cards, and the Caterpillar game, a novel set-matching game that did not require explicit counting). Children’s performance on the subsequent Mr. Elephant task may have been bolstered by practice effects, particularly experience with the Caterpillar game, which was similar to Mr. Elephant in that it tested children’s set representation skills and did not require explicit counting. In contrast, children in the current study had not participated in any game similar to Mr. Elephant. Also, in Shusterman et al.’s (2017) study, the experimenter started each trial using their finger to gesture to the set, whereas the current study did not do this. This may have made quantity more salient to the child when doing the task. Next, the sample sizes of the two studies were very different. Shusterman et al. (2017) analyzed performance in 9 subset knowers and 8 cardinal principle knowers, whereas the current study analyzed performance in 25 non-CP knowers and 120 cardinal principle knowers. It is possible that Shusterman et al.’s (2017) small sample size may not be as generalizable, especially when comparing to a larger and more linguistically diverse sample as in the current study. Finally, the two studies coded data differently. Shusterman et al. (2017) analyzed the percentage of correct responses on the two small trials, while the current study coded performance as a binary variable, either the participants answered both small quantity trials correctly or they did not, a stricter criterion.

### Possible Influence of Executive Functioning

When children must track objects that are temporarily hidden, working memory may be an important factor. Visual working memory capacity has been shown to improve as children develop with marked improvements to capacity from age 3 to 8 (Pailian et al., 2016) and with substantial changes in small set size capacity in children age 1 to 4 (Kibbe & Applin, 2022). Specifically with regard to small quantity representation, subitizing may require more attentional resources than large quantity estimation (Burr et al., 2010). Both working memory and language are associated with many early mathematical skills (e.g., Purpura & Ganley, 2014). Research on children with specific language impairment show deficits in both language comprehension and working memory (e.g., Archibald & Gathercole, 2006; Montgomery et al., 2010), strengthening claims that they are related skills. Perhaps children with better working memory skills perform better on the Mr. Elephant task, helping them keep in mind how many balls entered and how many exited. Similarly, other executive functioning skills could have also influenced performance on the task. Later access to language has been shown to negatively influence executive functioning abilities (Dye & Hauser, 2014; Goodwin et al., 2022; M. L. Hall et al., 2017; Marshall et al., 2015). Specifically, DHH children with later access to language performed worse on a nonverbal working memory task, while DHH children with signing deaf parents (language exposure starting from birth) performed equivalently to typically developing hearing children (Marshall et al., 2015). Critically, it may be difficult to separate the effects of Language Timing from executive functioning. An important next step would be to see if measures of executive functioning predict performance on Mr. Elephant.

Working memory may also be implicated given the sequential nature of the task (i.e., the balls being fed to Mr. Elephant sequentially, instead of all at once). Mix (1999b) found that sequentially presented sets were more difficult for children to represent compared to static sets. Children could recognize equivalence for static sets on average by age 3, but it took until age 3.5 for them to recognize sequentially presented sets of items and age 4 for sequentially presented events. Additionally, the ability to match sequential sets of items emerged only after children had at least minimal counting proficiency. Without foundational early number knowledge, such as small quantity representation, the cognitive load of representing sequential sets may make the task more difficult. This aligns with Spaepen and colleagues’ (2011) findings that homesigners, who do not have a count system, struggled on matching sequentially presented knocks.

### Influence of Socioeconomic Status on Language and Number

Our first model found that, in addition to Timing, SES also significantly predicted performance on Mr. Elephant; however, this effect disappeared in our second model which included Give-a-Number and Highest Count. This finding parallels results reported in Slusser et al. (2019), in which Give-a-Number fully mediated the relationship between parent education (a key component of family SES) and mathematical achievement one year later. In both accounts, the relationship between SES and mathematical outcomes was mediated by children’s Give-a-Number performance.

SES has been linked to mathematics performance (e.g., Jordan & Levine, 2009) and when SES was controlled, the amount of caregiver number talk predicted children’s number word knowledge (Levine et al., 2010). While the current study did not directly assess parental number talk, we did address Timing of language exposure. The Early and Later Language groups had systematically different access to language in their environments; since the Later Language group had reduced access to all language, it follows that they received less parental number talk as a result of having less language exposure in general. The results from our second model in which SES is no longer significant are consistent with previous literature that access to number talk may outweigh or mediate the effects of more distal factors like SES (Levine et al., 2010; Slusser et al., 2019).

Additionally, the groups exhibited major disparities in SES; while possible scores ranged from 3 to 66, the Later ASL group had a mean SES of 36 while the Early English group had a mean SES of 54. Although language modality was not a predictor of Mr. Elephant performance, English-users had significantly higher mean SES than ASL-users. Similarly for Timing of language exposure, a significant predictor of Mr. Elephant performance, Early-exposed children had higher mean SES than Later-exposed children. Given these group differences, it may be difficult to completely disentangle SES from other variables in this study and future work should address this. Note that although parent education level (one component of SES measurement) has a strong relationship to DHH children’s language outcomes, the presence of early intervention mediates that relationship (Chen & Liu, 2021). Nevertheless, it does appear that children’s language experiences, especially the timing of their first exposure to language, is the most important factor.

### How Might Later Access to Language Influence Small Quantity Representation?

The findings that language, specifically timing of first language exposure and cardinal principle knowledge, predicted small quantity representation performance was surprising and raises the question of how language may be exerting these effects. Here we speculate on possible mechanisms. First, language is a conceptual resource that helps the formation of categories (e.g., Fulkerson & Waxman, 2007). Perhaps CP-knowers get more practice classifying sets by number, so that even when they do not have to count, they still have this linguistically defined category as a resource (e.g., “three”) which could aid performance on the Mr. Elephant task. Language may enrich representations of small quantities (see Carey, 2009 for more discussion on enriched parallel individuation). However, current research has not yet investigated the consequences of delayed access to language on the enriched parallel individuation pathway and how later exposure to language might influence the nonverbal process of learning “setness” as a concept.

Additionally, language helps direct attention, memory and encoding (e.g., Altmann & Kamide, 2007; Finkbeiner et al., 2002); all of which are important for a task like Mr. Elephant in which children must track balls as they are presented, are occluded, and then reappear. Finally, there may be different developmental trajectories for small quantity representation abilities based on timing of first language exposure. Perhaps children who have delayed access to their first language follow a different developmental path than do typically developing children who receive language input starting at birth.

## CONCLUSION

Current theories and empirical work about the nature of humans’ representations of numerical magnitude suggest that linguistic knowledge is not necessary to track small quantities of objects. These results, drawn from a relatively rare population who experience unusual variability in their language experiences, suggest that the timing of language exposure and number word knowledge relate to the ability to track small quantities. More research is needed to investigate exactly how language may influence small quantity representation. Future research should explore completely nonlinguistic approaches to measuring quantity representation skills and consider the roles of other cognitive skills such as executive functioning in explaining the relationship between language experience and small quantity representation.

## Acknowledgments

This work was supported by the National Science Foundation [grant number 1553589], the Connecticut Institute for the Brain and Cognitive Sciences, and the Harriott Fellowship. We thank all members of the Language Creation Lab, as well as Clifton Langdon, Corina Goodwin, and Sierra Eisen on feedback on prior drafts. Thank you to Tom Castelli of the Wesleyan Machine Shop for helping to design and build the Mr. Elephant apparatus. Finally, we would like to thank participants’ families, local schools, libraries, and after-school programs for their help and involvement.

## Author Contributions

M.Q.: Data curation; Formal analysis; Visualization; Writing – original draft; Writing – review & editing. E.C.: Formal analysis; Writing – review & editing. K.W.: Data curation; Formal analysis; Writing – review & editing. A.S.: Conceptualization; Writing – review & editing. M.C.: Conceptualization; Funding acquisition; Resources; Supervision; Writing – review & editing.

## Funding Information

This work was supported by the National Science Foundation [grant number 1553589], the Connecticut Institute for the Brain and Cognitive Sciences, and the Harriott Fellowship.

## Data Availability Statement

The primary video data cannot be shared because it contains identifying information. Deidentified processed data is available on OSF (https://osf.io/mn5g6/?view_only=ecd90ffd239d4a15afc2356ece325dbe). A link to the analysis scripts on GitHub is also available on OSF.

## Notes

^1^ The Early ASL and Early English groups did not significantly differ in their Mr. Elephant performance (*X*^2^ (1, *N* = 84) = 0.243, *p* > 0.6), nor did the Later ASL and Later English groups (*X*^2^ (1, *N* = 70) = 0.810, *p* > 0.3).^2^ The mean age for CP-knowers who were early language-exposed was 5.2 years (*SD* = 0.90) and those who were later language-exposed was 5.5 years (*SD* = 0.92). The mean age for non-CP-knowers who were early language-exposed was 4.0 years (*SD* = 0.95) and those who were later language-exposed was 5.0 years (*SD* = 0.92). Carrigan et al. (in prep) found that chronological age and age of first exposure to language (but not modality) significantly predicted cardinal principle achievement, but when Highest Count was added to the model, the effect of age of exposure no longer reached significance.

## APPENDIX

**Table A1.** Mr. Elephant Trials. Small quantity trials analyzed are **bolded**.

	*Training Trial 1*	*Training Trial 2*	*Trial 1*	***Trial 2***	***Trial 3***	*Trial 4*	*Trial 5*	*Trial 6*
*# of Balls IN*	1	1	5	**3**	**2**	6	4	7
*# of Balls OUT*	1	0	4	**2**	**2**	6	4	6
